# Discrete regenerative fuel cell reduces hysteresis for sustainable cycling of water

**DOI:** 10.1038/srep04592

**Published:** 2014-04-04

**Authors:** Kiwon Park, Jungkoo Lee, Hyung-Man Kim, Kap-Seung Choi, Gunyong Hwang

**Affiliations:** 1 Department of Mechanical Engineering & High Safety Vehicle Core Technology Research Center, INJE University, 607 Eobang-dong, Gimhae-si, Gyongsangnam-do 621-749, Republic of Korea; 2 Department of Green Automobile Engineering, Youngsan University, 288 Junam-dong, Yangsan-si, Gyongsangnam-do 626-790, Republic of Korea; 3 Department of Automobile Engineering, Tongmyong University, 428 Sinseon-ro, Nam-gu, Busan, 608-711, Republic of Korea

## Abstract

The discrete regenerative fuel cell is being developed as a residential power control that synchronizes with a renewables load which fluctuates significantly with the time and weather. The power of proton exchange membrane fuel cells can be scaled-up adjustably to meet the residential power demand. As a result, scale-ups from a basic unit cell with a 25 cm^2^ active area create a serpentine flow-field on an active area of 100 cm^2^ and take into account the excessive current and the remaining power obtained by stacking single cells. Operating a fuel cell utilising oxygen produced by the electrolyser instead of air improves the electrochemical reaction and the water balance. Furthermore, the performance test results with oxygen instead of air show almost no hysteresis, which results in the very stable operation of the proton exchange membrane fuel cell as well as the sustainable cycle of water by hydrogen and oxygen mediums.

Proton exchange membrane (PEM) fuel cells were first employed in the Gemini space programme^1^. In the 1970s, the development of Nafion® led to the large-scale use of this membrane in fuel cells^2^,^3^,^4^. The use of direct fuels, particularly hydrogen (H_2_), is one of several possible long-term approaches to the improvement of energy efficiency, energy sustainability, energy security, greenhouse gas reduction and global environmental conservation^5^,^6^,^7^,^8^. The PEM fuel cell is a fuel cell technology dedicated to delivering electric loads that are used irregularly and that exhibit high transient dynamics and frequent start and stop operations. Nevertheless, the main disadvantages of fuel cells are derived from the cells' erratic behaviour due to the high sensitivity of the stack state.

The primary motivation for developing a DRFC system linked with PV arrays was to create a platform for examining the stand-alone load-regulating characteristics in a controlled environment^6^,^9^,^10^. The dynamic behaviour of fuel cells is an integral part of the overall stability and performance of power systems. Moreover, many applications require dynamic transient responses that are faster than those exhibited by fuel cell systems^11^. This discrepancy has significant implications on stack performance due to temporal oxidant starvation caused by poor air flow dynamics, which thereby leads to low efficiency^12^ and a low lifetime as a result of cathode surface area loss^13^.

In this study, we characterised the hysteresis effects of a scaled-up PEM fuel cell for dynamic operation applications in a discrete regenerative fuel cell (DRFC) system with H_2_ and oxygen (O_2_). An optimisation process for serial and parallel scale-ups of PEM fuel cells was developed. Detailed data regarding the effects of hysteresis on the polarisation and power density of a serial scaled-up stack and a parallel scaled-up single cell and on the H_2_/air and H_2_/O_2_ reactants are presented. Operating a fuel cell utilising O_2_ produced by an electrolyser instead of air improves the electrochemical reaction and the water balance. Furthermore, the performance test results with O_2_ instead of air show almost no hysteresis, which results in the very stable operation of fuel cells as well as the sustainable cycling of water by H_2_ and O_2_ media. The results of this study can help us advance the hybrid O_2_-DRFC system linked with photovoltaic (PV) arrays and supercapacitors for developing a sustainable smart grid.

## Results

To evaluate the performance and hysteresis of PEM fuel cells, we fabricated a basic unit cell, a serial scaled-up 4-cell stack and a parallel scaled-up single cell (Supplementary Fig. S1a) which featured novel serpentine flow channels combined with sub-channels and by-passes (Supplementary Fig. S1b and Table S1)^14^,^15^,^16^,^17^,^18^. The properties of a membrane electrode assembly (MEA), which is composed of two gas diffusion layers (GDLs), a membrane and two electrodes, are presented in Supplementary Table S2^15^. The fuel cell performance was evaluated by the polarisation curve measured while adjusting the pressure, temperature, humidity, and flow rate of the reacting gas using the test equipment (Supplementary Fig. S2 and Supplementary Table S3). We tested the polarisation and power density of the basic unit cell, the serial scaled-up 4-cell stack and the parallel scaled-up single cell utilising air and O_2_ as an oxidiser over 10 cycles while the load currents were controlled according to predefined schedules (Supplementary Fig. S3). Although the polarisation and power density curves with air and O_2_ as an oxidiser over 10 cycles were slightly different, such reproducible results were attributed to the change in the membrane conductivity, which caused the water content to move towards a higher current density zone^19^,^20^ (Supplementary Fig. S4). Detailed data regarding the hysteresis loop areas for the polarisation and power density of the basic unit cell, the serial scaled-up 4-cell stack and the parallel scaled-up single cell utilising air and O_2_ over 10 cycles are presented (Supplementary Table S4).

**Figure 1:**
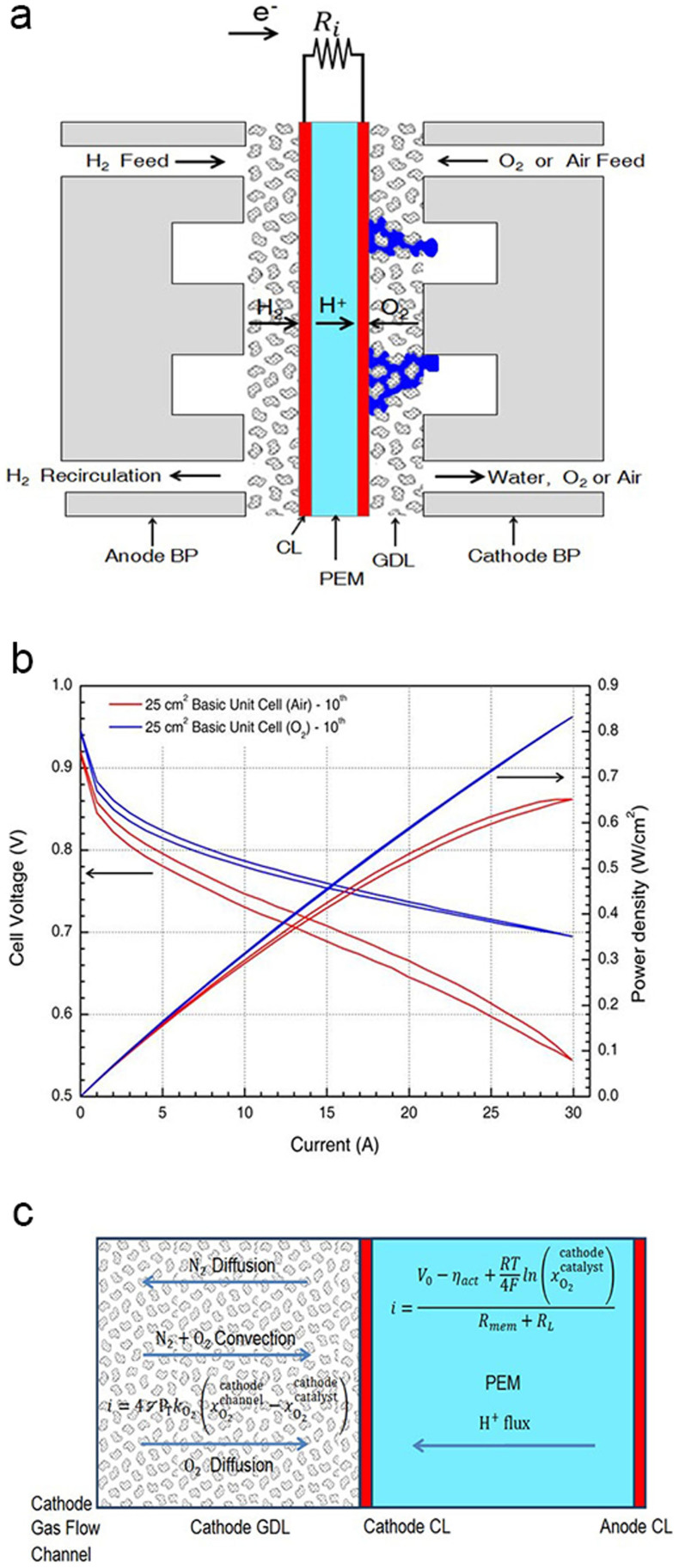
Characterisation of the hysteresis effect in a basic unit cell using H_2_/air and H_2_/O_2_ reactants. (a) Schematic of mass and charge transport in a PEM fuel cell. Oxygen and hydrogen flow through gas flow channels in the bipolar plates at the cathode and anode, respectively. The O_2_ and H_2_ must be transported through the GDLs to the respective CLs, where the electrochemical reactions occur. (b) Comparisons of the polarisation and power density curves at the 10th cycle for basic unit cells utilising H_2_/air (red) and H_2_/O_2_ (blue). (c) Schematic of a PEM fuel cell with transport resistances across the cathode GDL and the PEM^20^. Oxygen is consumed by a reaction at the CL, driving both convective and diffusive flows across the GDL. The convection is driven by a pressure difference resulting from a reduction in the molar concentration associated with oxygen consumption at the catalyst. The diffusion across the cathode GDL is driven by the concentration difference between the gas flow channel and the CL. The proton current is driven by the chemical potential difference between the chemical potential of hydrogen at the anode and cathode CLs.

The hysteresis of polarisation curves can be observed during the repetitive increase and decrease in input current. The lower curves of hysteresis loops are created as the input current increases, and the upper curves are created as the input current decreases. These phenomena are related to the flooding and drying of the fuel cell, respectively. If flooding occurs on the cathode side, then operation at a higher current would only make the situation worse because additional water would be produced (Fig. 1a). The polarisation curve recorded under a decreasing current would show a lower voltage at high currents than the polarisation curve recorded under an increasing current^14^. The polarisation and power density curves for a basic unit cell exhibit varying degrees of hysteresis; notably, the degrees of hysteresis with H_2_/O_2_ are lower than those with H_2_/air (Fig. 1b). We arrange the data of the hysteresis loop areas over 10 cycles with mean and standard error of the mean (s.e.m.). The dynamic stability is quantitatively explained by the hysteresis loop areas over 10 cycles with H_2_/air (polarisation curve: 0.4330 ± 0.0082 (mean ± s.e.m.); power density curve: 0.2652 ± 0.0051) and H_2_/O_2_ (polarisation curve: 0.1510 ± 0.0014 (mean ± s.e.m.); power density curve: 0.0703 ± 0.0014). These results indicate that the cathode flooding with H_2_/air increases the O_2_ mass transfer resistance in the electrode and the degrees of hysteresis in the polarisation and power density with H_2_/air become worse than those with H_2_/O_2_. In case of air, O_2_ is transported across the cathode GDL by a combination of pressure-driven convection through the porous GDL and concentration-driven diffusion, while N2 is transported in the opposite direction across the cathode GDL by a concentration-driven diffusion (Fig. 1c). Because the vapour in the cathode gas flow channel is saturated with water vapour, additional water produced at the cathode must be immediately condensed.

**Figure 2:**
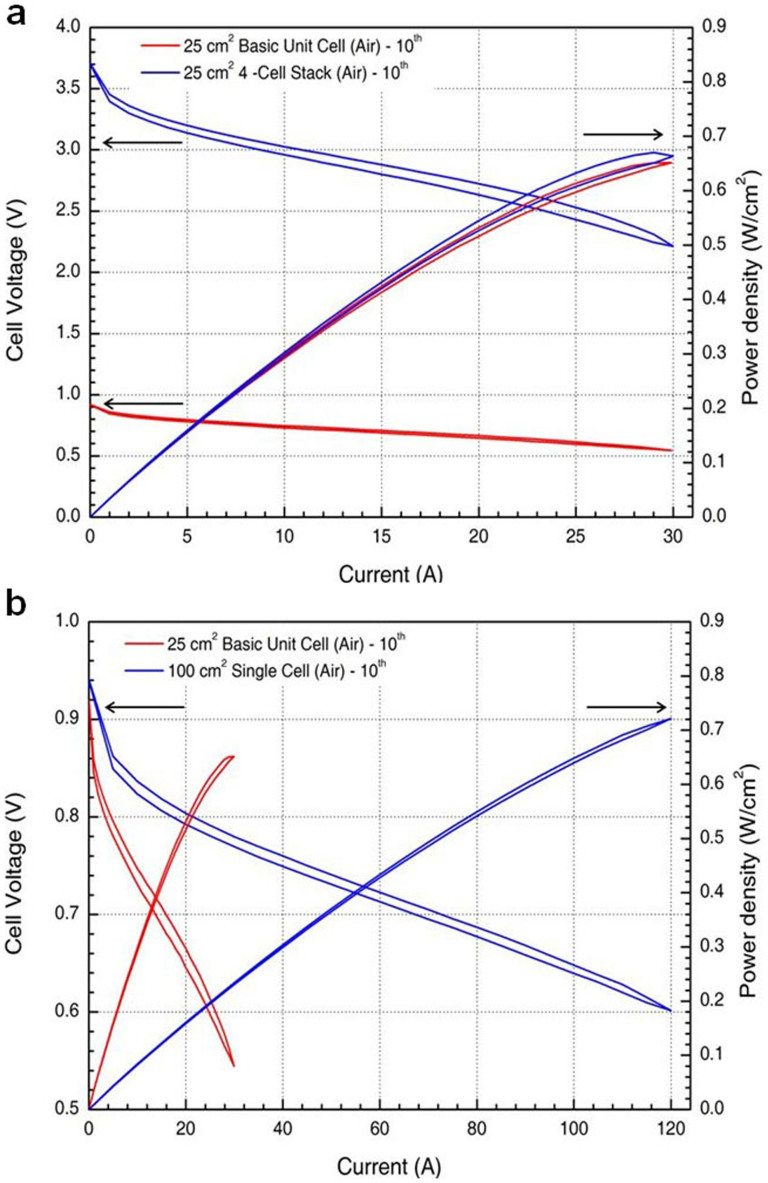
Optimised structures and performances of the parallel scaled-up single cell and the serial scaled-up 4-cell stack with reactants of H_2_/air. (a) Comparisons of the polarisation curve and the power density curve at the 10^th^ cycle between the basic unit cell (red) and the serial scaled-up single cell (blue) with reactants of H_2_/air. (b) Comparisons of the polarisation curve and the power density curve at the 10^th^ cycle between the basic unit cell (red) and the parallel scaled-up single cell (blue) with reactants of H_2_/air.

Fuel cell stacks consist of single cells connected in series or in parallel depending on the voltage and current requirements for specific applications^21^. These single cells experience some loss of efficiency and power density because of the scale-up in the area of the electrodes and the increased number of cells in the stack. These maldistribution problems could be reduced if the manifold of a parallel gas distribution system was optimised to homogenise the gas distribution within the stack^22^. Recently, the convection-enhanced serpentine flow-field design has been confirmed to exhibit a better water-handling ability than the conventional design^15^,^16^,^17^, and the cathode flow-field design for a single serpentine PEM fuel cell has promoted strong convection flows to enhance O_2_ transport and water removal^23^.To optimise the scale-up of the basic unit cell over a 25 cm^2^ active area, fuel cells with a total active area of 100 cm^2^ scaled up in series and in parallel were experimentally studied (Fig. 2 and Supplementary Fig. S1a).

The power density of the serial scaled-up stack was slightly higher than that of the basic unit cell with reactants of H_2_/air (Fig. 2a); this discrepancy was caused by the optimisation of the water management conditions near the higher current density zone because the stack was sufficiently cooled after exposure to electrochemical reaction heat. Therefore, the serial scale-up achieved by stacking single cells requires the careful design of a cooling system to improve the performances of the fuel cell stack. In this study, maximum cell voltage of the serial scaled-up stack was approximately four times higher than that of the basic unit cell; therefore, we could safely stack the single cells despite the complexities of the interconnected system. The hysteresis loop areas over 10 cycles were quantitatively evaluated for the serial scaled-up stack design with reactants of H_2_/air (polarisation curve: 2.2097 ± 0.0228 (mean ± s.e.m.); power density curve: 0.3661 ± 0.0048).

The power density of the parallel scaled-up single cell was higher than that of the basic unit cell with reactants of H_2_/air (Fig. 2b). The main reason for this discrepancy is that the removal of the product, liquid water, becomes more difficult in larger systems; however, a high water vapour pressure in the reactant flow causes an increase in the overpotential, especially at the cathode^24^. These maldistribution problems can be reduced if the manifold of a parallel gas distribution system is optimised to homogenise the gas distribution. The maximum cell currents of the parallel scaled-up single cell and the basic unit cell were limited to 120 A and 30 A, respectively. Therefore, parallel scaled-up single cells should be limited under heavy current loads for safety and compactness in DRFC systems. The hysteresis loop areas over 10 cycles were quantitatively evaluated using the serial scaled-up stack with reactants of H_2_/air (polarisation curve: 0.2751 ± 0.0114 (mean ± s.e.m.); power density curve: 0.0335 ± 0.0010).

**Figure 3:**
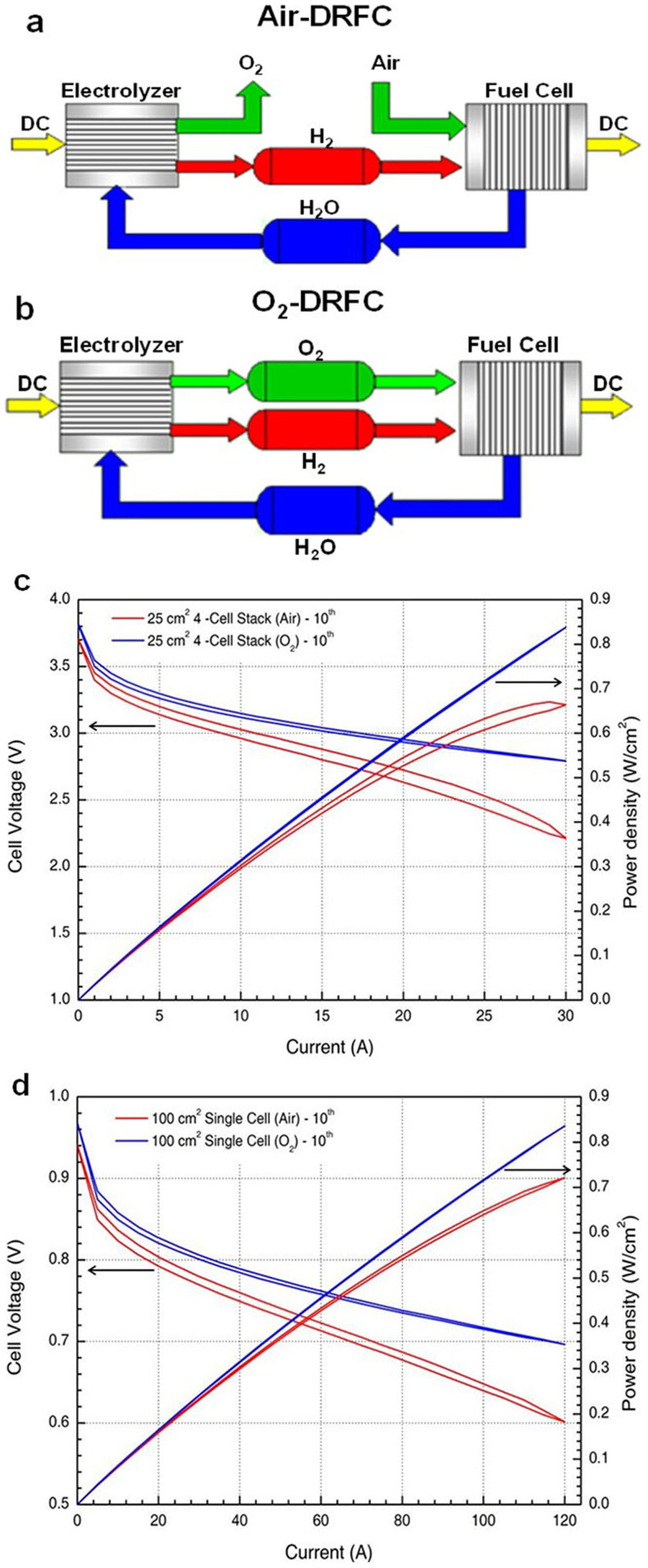
Performance enhancements achieved by utilising pure oxygen from the PEM electrolyser instead of air. (a) Schematic of an air-DRFC consisting of a PEM electrolyser and a PEM fuel cell utilising H_2_/air; the oxygen produced in the electrolysis mode is purged to the atmosphere. (b) Schematic of an O_2_-DRFC consisting of a PEM electrolyser and a PEM fuel cell utilising H_2_/O_2_; the pure oxygen produced in the electrolysis mode is supplied to the PEM fuel cell. (c) Comparisons of the polarisation and power density curves at the 10^th^ cycle with the serial scaled-up 4-cell stack on an active area of 5 × 5 cm^2^ between the air-DRFC (red) and the O_2_-DRFC (blue). (d) Comparisons of the polarisation and power density curves at the 10^th^ cycle with the parallel scaled-up single cell on an active area of 12.5 × 8 cm^2^ between the air-DRFC (red) and the O_2_-DRFC (blue).

Scaled-up PEM fuel cells in air-DRFC (Fig. 3a) and O2-DRFC (Fig. 3b) configurations have been developed^25^. O_2_-DRFC systems exhibit higher performance and efficiency, do not require oxidant compressors and can operate in a completely closed cycle with little maintenance but require approximately 50% more tank space to store O_2_, which can be a safety hazard due to its high flammability. The operation of a fuel cell with O_2_ instead of air using a platinum catalyst supported on carbon yields a 17% improvement in fuel cell efficiency^9^. Consequently, O_2_-DRFC systems are currently preferred in cases in which compressed O_2_ storage does not pose a significant safety hazard.

We present the experimental results obtained in this study by comparing the polarisation and the power density curves between the reactants of H_2_/O_2_ and H_2_/air in the serial scaled-up 4-cell stack and the parallel scaled-up single cell, respectively (Fig. 3c, d). In the serial scaled-up 4-cell stack, the areas of the hysteresis loops over 10 cycles were quantitatively evaluated with H_2_/O_2_ (polarisation curve: 0.7520 ± 0.0834 (mean ± s.e.m.); power density curve: 0.0906 ± 0.0058). The maximum power densities with the H_2_/air and H_2_/O_2_ reactants were 0.664 W/cm^2^ and 0.838 W/cm^2^, respectively; therefore, the reactants of H_2_/O_2_ exhibited 26.2% higher power density. In the parallel scaled-up single cell, the areas of the hysteresis loops over 10 cycles were quantitatively evaluated with H_2_/O_2_ (polarisation curve: 0.1505 ± 0.0131 (mean ± s.e.m.); power density curve: 0.0167 ± 0.0018). The maximum power densities with the H_2_/air and H_2_/O_2_ reactants were 0.722 W/cm^2^ and 0.836 W/cm^2^, respectively; therefore, the reactants of H_2_/O_2_ exhibited 15.8% higher power density. The dynamic stability and performance of the fuel cells were confirmed to be higher with H_2_/O_2_ than with H_2_/air in both the serial scaled-up 4-cell stack and the parallel scaled-up single cell.

## Discussion

The PEM fuel cells utilised in the experiments featured serpentine flow channels combined with sub-channels and by-passes. Previous studies have shown that the implementation of a novel flow-field design improves not only the flow consistency of the inner reaction gas and the liquid water behaviour but also the current density in the ohmic resistance loss region and mass transport loss region. Compared to the conventional flow-field design, the novel flow-field design features a much smaller area surrounded by the hysteresis curve because of a varying electric load, but no difference in the hysteresis curve is observed in the high-current-density region. To demonstrate the dynamic behaviours of the basic unit cell with the novel flow-field design, which features added sub-channels and by-passes with respect to the conventional flow-field design, we investigated the performance of the polarisation and power density curves of cells with H_2_/air and H_2_/O_2_ reactants.

The hysteresis effects of PEM fuel cells were investigated under serial and parallel scale-up designs by increasing the active area, stacking single cells and enhancing the power generated by utilising H_2_/O_2_ instead of air as a reactant. Thus, we could observe the hysteresis effects created in the fuel cell systems as the load was fluctuating. When the membrane of a PEM fuel cell is fully hydrated, the water is balanced within the MEA^26^. A larger void volume in the diffusion layer is most likely to be occupied by liquid water when using pure O_2_. The fuel cell can run at a higher current density with a small degree of gas diffusion if the generated water is carried out of the fuel cell by the gas. Thus, the hysteresis in the polarisation curves of O_2_-DRFC systems is reduced, allowing for the sustainable cycling of water.

## Methods

### Hysteresis characterisation

PEM fuel cell performance was evaluated by polarisation curves measured while adjusting the pressure, temperature, humidity, flow rate and reacting gas using fuel cell test equipment built at INJE University. Experiments were carried out using the FCTESTNET (Fuel Cells Testing & Standardisation NETwork) performance test procedure^27^, which was originally developed under the Research & Training Network (RTN). The FCTESTNET performance test procedure is a method used to evaluate the activation loss region and the ohmic resistance loss region of fuel cells. In the performance tests, the load current was controlled to minimise physical damage to the MEA and to maintain a stable electrical load. The detailed test procedures are as follows: for the experiments with the 25 cm^2^ basic unit cell and the 25 cm^2^ serial scaled-up stack, the cell current was increased from 0 A to 30 A in increments of 1 A and then decreased to 0 A in decrements of 1 A for 300 s, whereas for the experiments with the 100 cm^2^ parallel scaled-up stack, the cell current was increased from 0 A to 120 A in increments of 5 A and then decreased to 0 A in decrements of 5 A for 500 s.

To analyse the hysteresis characteristics quantitatively, a mathematical integration programme that calculates the area inside the hysteresis loop in the polarisation and power density curves was developed using Matlab®. In the programme, the areas between upper and lower curves in a hysteresis loop were calculated by taking the integral of the polynomial equation of each curve from the lower limit to the upper limit of the current variations on the x-axis. Then, the area covered by the lower curve was subtracted from the area covered by the upper curve to obtain the hysteresis loop area.

### Preparation of fuel cell

Fuel in the form of H_2_ gas is fed into the anode side of the PEM fuel cell. The oxidant in a fuel cell is O_2_, either in air or as a pure gas, which enters the fuel cell through the cathode inlet. The reactant gases H_2_ and O_2_ flow through channels machined into graphite plates and migrate through GDLs to the catalyst surface on their respective sides of the MEA. Aided by the noble metal catalyst in the anode, hydrogen is oxidised to form protons and electrons. To evaluate the performance of PEM fuel cells, we fabricated a basic unit cell with 5 passes and 4 turns on an active area of 5 × 5 cm^2^, a serial scaled-up 4-cell stack with 5 passes and 4 turns on an active area of 5 × 5 cm^2^ and a parallel scaled-up single cell with 7 passes and 4 turns on an active area of 12.5 × 8 cm^2^.

### Fuel cell testing

The bipolar plates (BPs) of the anode and the cathode were produced in the same semi-counter flow format; that is, the inlet and the outlet of hydrogen were at the top left and the bottom right, respectively, while the inlet and the outlet of oxygen or air were at the top right and the bottom left, respectively. A unique feature of PEM fuel cells compared with other types of fuel cells is that the former feature a solid proton-conducting electrolyte. The MEA is the ‘heart’ of the PEM fuel cell. In this study, a W.L. Gore & Associates PRIMEA® Series 57 MEA (anode: Pt catalyst 0.4 mg/cm^2^, cathode: Pt catalyst 0.4 mg/cm^2^) was used and sandwiched between anode and cathode SIGRACET® GDLs, which have a porous structure^28^. The MEA has a membrane and two electrodes composed of highly dispersed carbon-supported platinum catalysts. The PEM fuel cell performance was evaluated by measuring polarisation curves while adjusting the pressure, temperature, humidity, flow rate and reacting gas using fuel cell test equipment built at INJE University. Because the PEM fuel cell performance depends greatly on the operating conditions, the test equipment controlled the operation of the PEM fuel cells carefully and continuously. The test equipment consists of an electronic load, a flow controller, a temperature controller, a relative humidity controller and a DEWETRON DEWE-30-16 data acquisition system.

The initial performance of the fuel cells was greatly affected by the humidification of the MEA, the electrical load and the abundant supply of the reaction gas. Therefore, sufficient humidification was induced for 30 min to hydrate the dried MEA prior to measuring cell performance. The experiment was carried out using the FCTESTNET performance test procedure. The FCTESTNET performance test procedure is a method used to evaluate the activation loss region and the ohmic resistance loss region of fuel cells. In the performance test, the load current was controlled to minimise physical damage to the MEA and to maintain a stable electrical load. The results of each test procedure were confirmed by supplemental literature and experiments^15^,^16^,^17^.

## Author Contributions

Experiments were designed by K.-S.C. and H.-M.K., fieldwork was performed by K.P. and J.L., statistical analyses were performed by K.P. and H.-M.K., supplementary methods and analysis were performed by G.H., K.-S.C. and H.-M.K., and writing was performed by K.-S.C. and H.-M.K. with the input from K.P., J.L. and G.H. All authors discussed the results and commented on the manuscript.

## Supplementary Material

Supplementary Information Supplementary Information
