# Supplementary material for: Discrete regenerative fuel cell reduces hysteresis for sustainable cycling of water
Source: Sci Rep. 2014 Apr 4;4:4592. doi: 10.1038/srep04592 (PMC3975235; doi:10.1038/srep04592)
Supplement: Supplementary Information [file srep04592-s1.pdf]

Supplementary Information

Discrete regenerative fuel cell reduces hysteresis for sustainable cycling of water

Kiwon Park[2], Jungkoo Lee[1], Hyung-Man Kim[1*], Kap-Seung Choi[3] & Gunyong Hwang[2]

[1]Department of Mechanical Engineering & High Safety Vehicle Core Technology Research Center, INJE University, 607 Eobang-dong, Gimhae-si, Gyongsangnam-do 621-749, Republic of Korea

[2]Department of Green Automobile Engineering, Youngsan University, 288 Junam-dong, Yangsan-si, Gyongsangnam-do 626-790, Republic of Korea

[3]Department of Automobile Engineering, Tongmyong University, 428 Sinseon-ro, Nam-gu, Busan, 608-711, Republic of Korea

*Correspondence to: Hyung-Man Kim, mechkhm@inje.ac.kr

This file includes:

Figures S1 to S4

Tables S1 to S5

Supplementary Figure S1

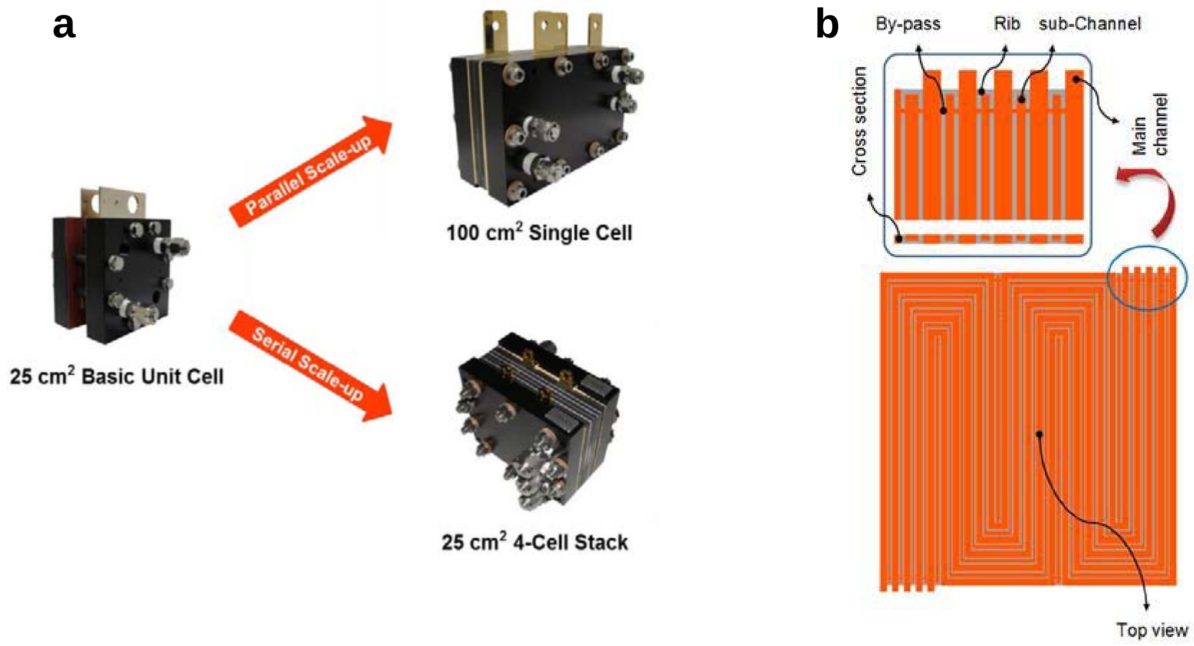

Figure S1. (a) Photograph of a parallel scaled-up single cell with 7 passes and 4 turns on an active area of 12.5 × 8 cm^2, and a serial scaled-up 4-cell stack with 5 passes and 4 turns on an active area of 5 × 5 cm^2 from the basic unit cell of 5 passes and 4 turns on an active area of 5 × 5 cm^2 (not to scale), and (b) the serpentine flow-field with sub-channels and by-passes [adapted from Ref. 15].

Supplementary Figure S2

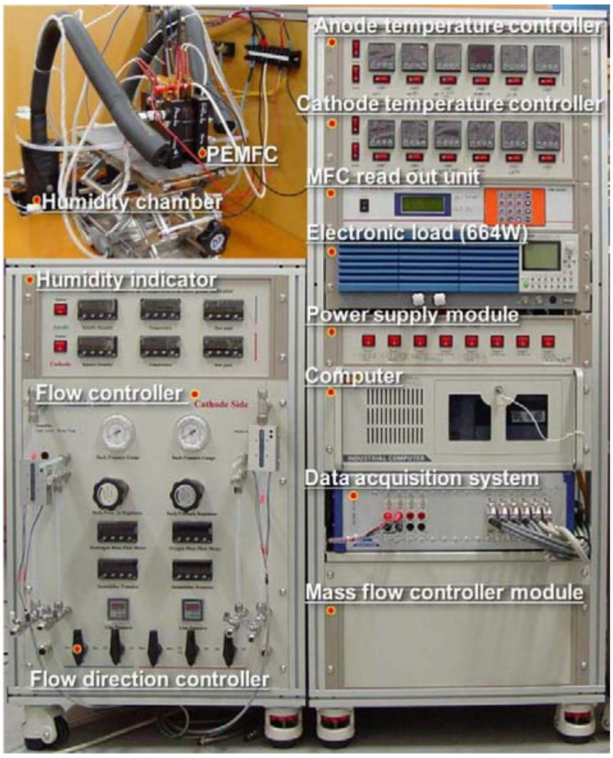

Figure S2. *Photograph of the fuel cell test equipment for control and measurement built at INJE University.* The performance evaluation of PEM fuel cells is done with potentiostatic and galvanostatic techniques using the measured polarisation curve while adjusting the operating pressure, temperature, humidity, flow rate and reacting gas. The custom designed test equipment is constructed with a flow direction controller, flow controller, temperature controller, humidity sensor module, mass flow controller, electronic load and data acquisition system to control the operating conditions continuously. The specifications of each device are listed in Supplementary Table S4.

Supplementary Figure S3

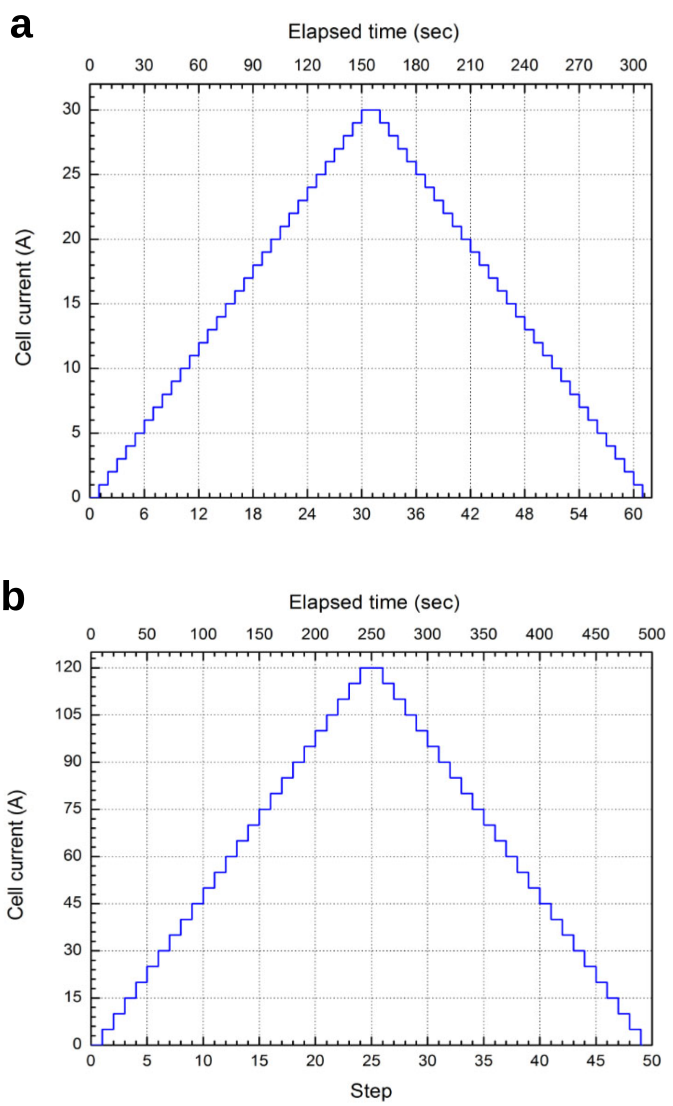

Figure S3. *FCTESTNET maximum performance test procedure of the PEM fuel cell developed by EU FP5*[28]. The maximum performance test procedure using the galvanostatic technique (CC mode) with the current increasing and decreasing (**a**) from 0 A to 30 A in decrements of 1 A (25 cm^2 basic unit cell and 4-cell stack) and (**b**) from 0 A to 120 A in decrements of 5 A (100 cm^2 single cell) has been processed with 10 s per step to find the performance from low current density to high current density.

Supplementary Figure S4

a

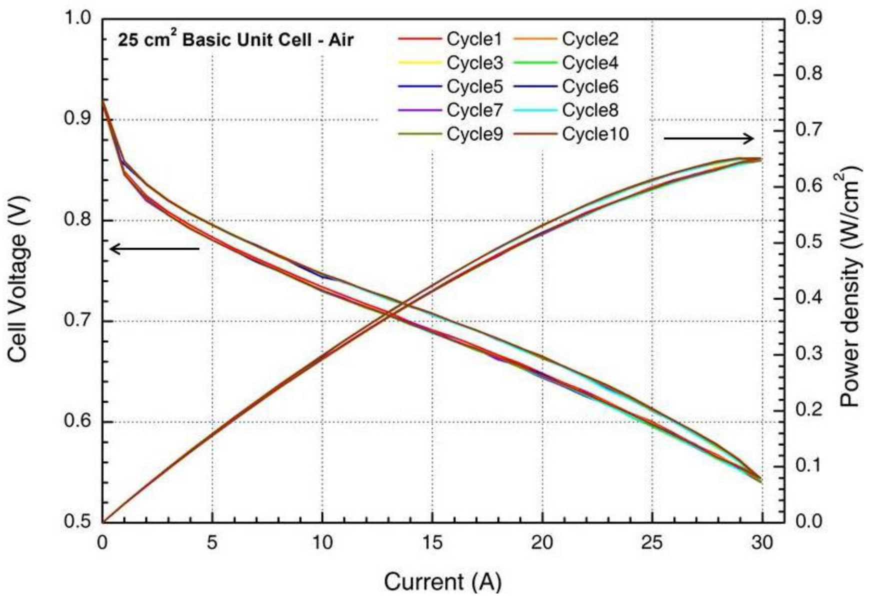

b

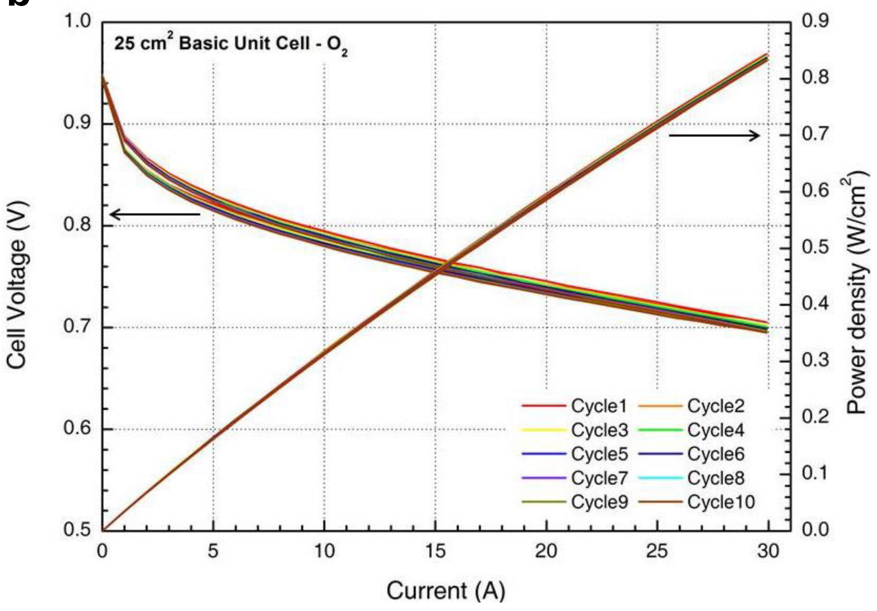

c

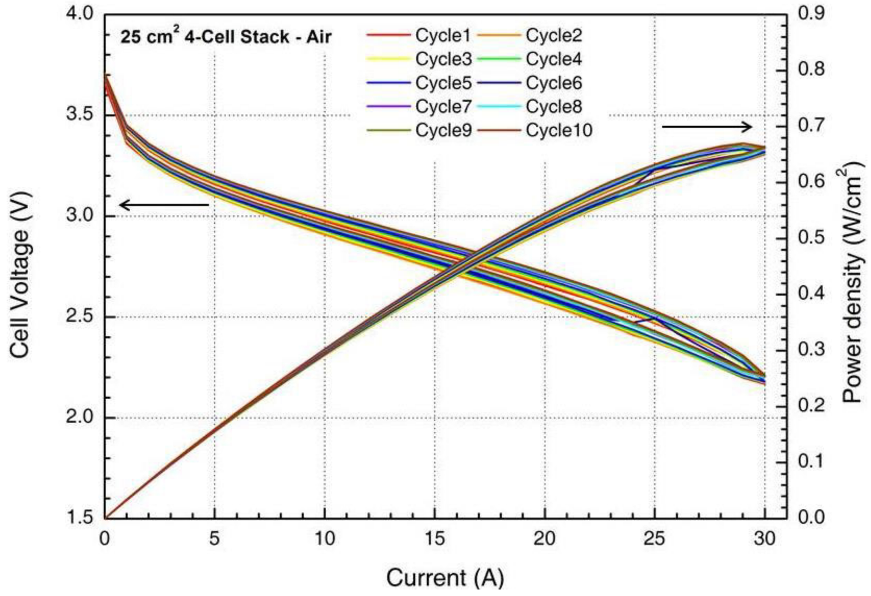
25 cm² 4-Cell Stack - Air
Cycle1
Cycle2
Cycle3
Cycle4
Cycle5
Cycle6
Cycle7
Cycle8
Cycle9
Cycle10
Cell Voltage (V)
Power density (W/cm²)
Current (A)
4.0
3.5
3.0
2.5
2.0
1.5
0.9
0.8
0.7
0.6
0.5
0.4
0.3
0.2
0.1
0.0
0
5
10
15
20
25
30

d

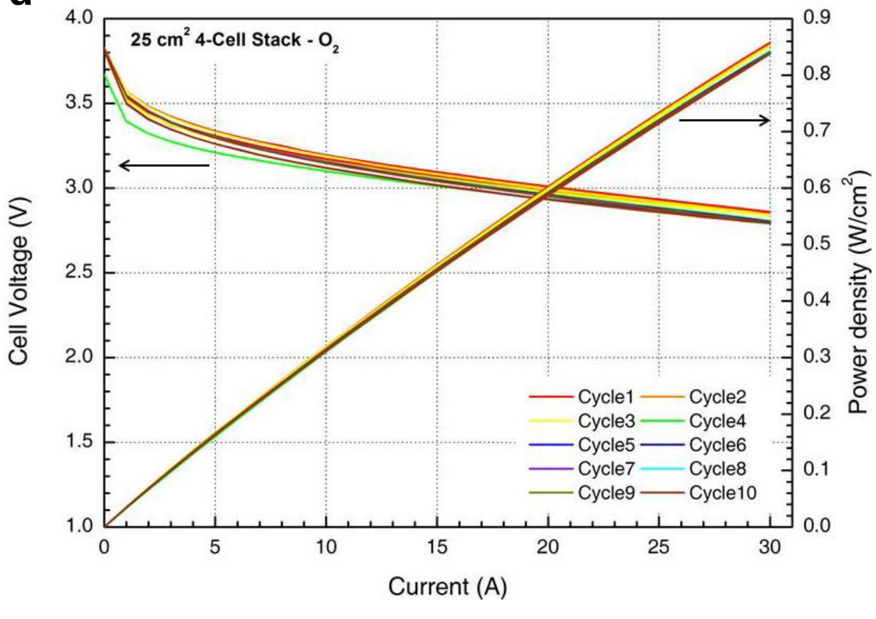
25 cm² 4-Cell Stack - O₂
Cycle1
Cycle2
Cycle3
Cycle4
Cycle5
Cycle6
Cycle7
Cycle8
Cycle9
Cycle10
Cell Voltage (V)
Power density (W/cm²)
Current (A)
4.0
3.5
3.0
2.5
2.0
1.5
1.0
0.9
0.8
0.7
0.6
0.5
0.4
0.3
0.2
0.1
0.0
0
5
10
15
20
25
30

e

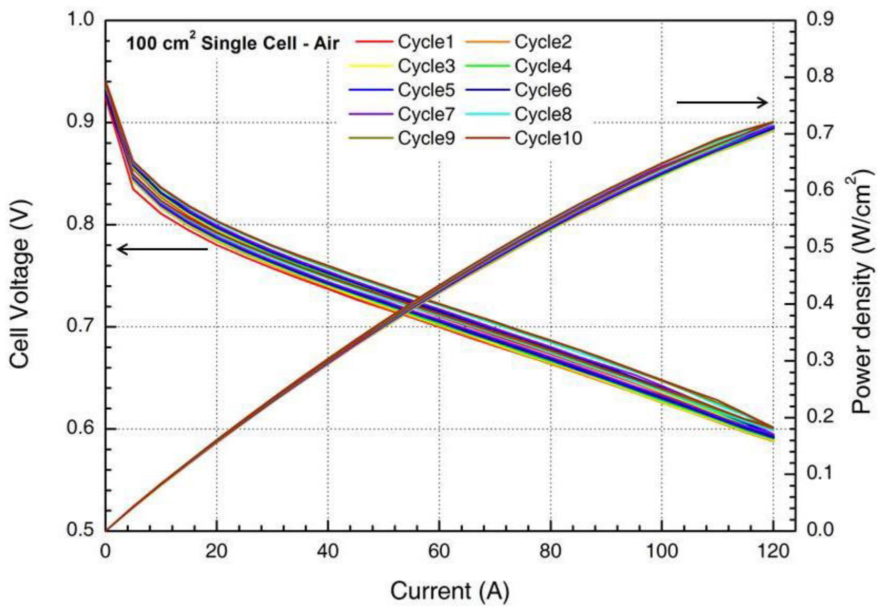

f

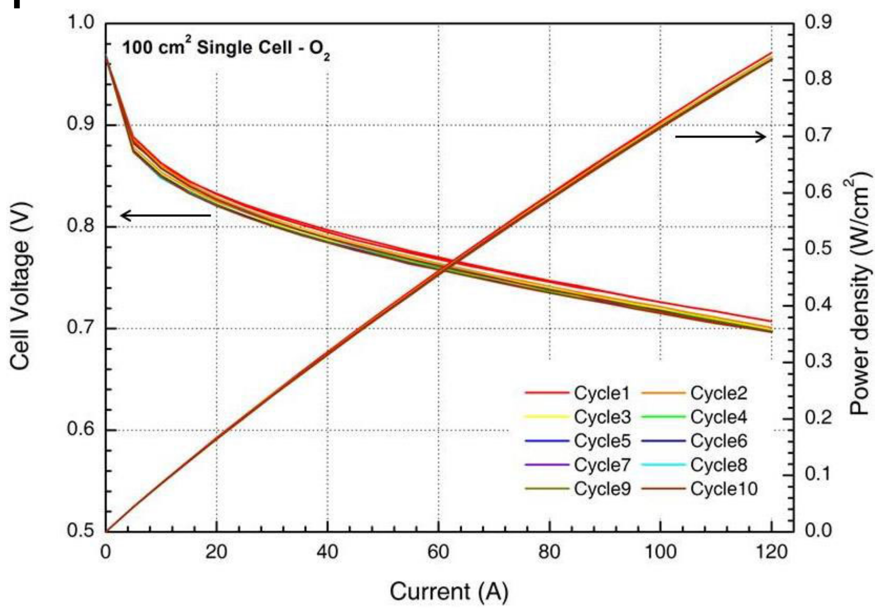

Figure S4. *A comparison of hysteresis effects in the polarisation and power density curves with the basic unit cell, the serial scaled-up 4-cell stack and the parallel scaled-up single cell*

utilising air and O_2 as an oxidiser during 10 cycles of the performance test procedure. (**a**) The polarisation and power density hysteresis curves for the basic unit cellutilising the reactants of H_2/air. (**b**) The polarisation and power den cell utilising the reactants of H_2/O_2. (**c**) The polarisation and power density hysteresis curves for the serial scaled-up 4-cell stack utilising the reactants of H_2/air. (**d**) The polarisation and power density hysteresis curves for the serial scaled-up 4-cell stack utilising the reactants of H_2/O_2. (**e**) The polarisation and power density hysteresis curves for the parallel scaled-up single cell utilising the reactants of H_2/air. (**f**) The polarisation and power density hysteresis curves for the parallel scaled-up single cell utilising the reactants of H_2/O_2.

Supplementary Table S1. Geometric details of the conventional advanced serpentine flow-field (CASFF) and the serpentine flow-field with sub-channels and by-passes (SFFSB) used in this study.

Flow-field patterns	CASFF	SFFSB
Main channel width (mm)	1.0	1.0
Main channel rib width (mm)	1.0	1.0
Main channel turn rib width (mm)	1.25	1.25
Main channel height (mm)	0.5	0.5
Sub-channel width (mm)	-	0.5
Sub-channel turn rib width (mm)	-	0.75
Sub-channel height (mm)	-	0.334
By-pass width (mm)	-	0.25
By-pass height (mm)	-	0.334
Cross-sectional area (cm^2)	0.025	0.025

Supplementary Table S2. Components and properties of the MEA used in this study.

MEA components and properties	Value
**Current collector**	
Thermal conductivity (W/m·K)	5.7
**GDL**	
Thickness after compressed (μm)	250
Permeability (m^2)	1.0 e-12
Porosity after compressed (%)	70
Diffusion adjustment (%)	50
Thermal conductivity (W/m·K)	0.21
**Membrane electrode assembly**	
Thickness (μm, including 12.5 μm thickness of catalyst layer)	50
Thermal conductivity (W/m·K)	0.15
Dry membrane density (g/cm^3)	2.0
Equivalent weight of the dry membrane (g/mol)	1100
Cathode exchange current density (A/cm^2)	0.02
Cathode transfer coefficient	0.6
Anode exchange current density (A/cm^2)	0.2
Anode transfer coefficient	1.2

Supplementary Table S3. Inlet conditions at the anode and cathode, as well as the operating conditions used in this study.

**Inlet conditions at the anode**		
Gas	H_2	
Stoichiometry	1.5	
Inlet temperature (℃)	75	
Inlet relative humidity (%)	100	
Mass fraction of H_2	0.078	
**Inlet conditions at the cathode**		
Gas	O_2	Air
Stoichiometry	1.2	2.0
Inlet temperature (℃)	70	70
Inlet relative humidity (%)	100	100
Mass fraction of O_2 and air	0.726	0.169
**Operating conditions**		
%H_2 in reformate	75	
Exit pressure (kPa)	101	
Open circuit voltage (V)	0.96	
Cell temperature (℃)	75	

Supplementary Table S4. Summary of the sensors monitoring and recording all needed data by the fuel cell test equipment.

**Description**	**Source**	**Sensor**	**Range**		**Quantity**
			**Signal**	**Physical**	
Cell voltage	PEM fuel cell	DC voltage	0 - 5 V	0-1.5 V	1
Cell current	Electronic load	DC voltage	0 - 5 V	0-50 A	1
Oxygen flowrate	KOFLOC 3660, 20 SLPM, O_2	DC voltage	0 - 5 V	0-5 SLPM	1
Air flowrate	KOFLOC 3660, 20 SLPM, Air	DC voltage	0 - 5 V	0-5 SLPM	1
Hydrogen flowrate	KOFLOC 3660, 20 SLPM, H_2	DC voltage	0 - 5 V	0-5 SLPM	1
Nitrogen flowrate	KOFLOC 3660, 20 SLPM, N_2	DC voltage	0 - 5 V	0-5 SLPM	1
Humidifier pressure	OMRON pressure sensor, 5 bar	DC voltage	4 - 20 mA	0-5 bar	2
Back pressure	OMRON pressure sensor, 5 bar	DC voltage		0-5 bar	2
Humidity sensor	VAISALA HMT337	-	-	-	2
Dew point	Warmed probe	DC voltage	0 - 5 V	-20~100℃	-

Temperature	PT 100Ω	DC voltage	0 - 5 V	-40~180℃	-
Relative humidity	Calculation	DC voltage	0 - 5 V	0-100 %RH	-
Water level sensor	At indicator	DC voltage	0 - 5 V	0-5 V (on/off)	2
Water temperature	At humidifier	PT 100 Ω	-	-	2
Gas outlet temperature	At humidifier	Type K	-	-	2
Humidity chamber temperature	At humidity chamber	Type K	-	-	2
Cell temperature	Attached to bipolar plate	Type K	-	-	2
Line temperature	At gas tube	Type K	-	-	4
Power source	SMPS (DC 5, 12, 24, 48 V)	DC voltage output	-	-	1

Supplementary Table S5. Summary of the areas of polarisation and power density curves for the basic unit cell, the serial scaled-up stack and the parallel scaled-up single cell utilising the H_2/air and H_2/O_2 reactants during 10 cycles by the mathematical integration programme.

Cycle	Reactants of H_2/air		Reactants of H_2/O_2	
	Polarisation	Power density	Polarisation	Power density
	Hysteresis areas produced with 25 cm^2 Basic Unit Cell			
1	0.3736	0.2328	0.1423	0.0605
2	0.4268	0.2564	0.1473	0.0686
3	0.4429	0.2732	0.1489	0.0685
4	0.4698	0.2908	0.1513	0.0716
5	0.4147	0.2538	0.1517	0.0706
6	0.4515	0.2787	0.1511	0.0722
7	0.4313	0.2593	0.1531	0.0716
8	0.4342	0.2673	0.1568	0.0751
9	0.4344	0.2643	0.1485	0.0683
10	0.4506	0.2751	0.1581	0.0759
mean	0.4330	0.2652	0.1510	0.0703
s.e.m	±0.0082	±0.0051	±0.0014	±0.0014
Cycle	Hysteresis areas produced with 25 cm^2 4-Cell Stack			
1	2.0823	0.3502	0.5074	0.0661
2	2.3252	0.3840	0.6550	0.0817

3	2.2660	0.3731	0.7004	0.0864
4	2.2649	0.3775	1.4785	0.1369
5	2.2588	0.3766	0.7633	0.0981
6	2.1324	0.3319	0.7150	0.0911
7	2.1743	0.3626	0.6933	0.0884
8	2.2217	0.3729	0.6809	0.0870
9	2.1895	0.3669	0.6688	0.0856
10	2.1820	0.3655	0.6577	0.0842
mean	2.2097	0.3661	0.7520	0.0906
s.e.m	±0.0228	±0.0048	±0.0834	±0.0058

**Cycle**	**Hysteresis areas produced with 100 cm^2 Single Cell**			
1	0.3728	0.0417	0.0483	0.0024
2	0.2541	0.0313	0.1287	0.0128
3	0.2489	0.0306	0.1340	0.0150
4	0.2495	0.0306	0.1518	0.0172
5	0.2575	0.0317	0.1551	0.0180
6	0.2718	0.0320	0.1603	0.0185
7	0.2810	0.0351	0.1679	0.0197
8	0.2729	0.0341	0.1854	0.0210
9	0.2709	0.0340	0.1939	0.0210
10	0.2724	0.0344	0.1795	0.0209
mean	0.2751	0.0335	0.1505	0.0167

s.e.m	±0.0114	±0.0010	±0.0131	±0.0018
